# MAGNITIVE: Effectiveness and Feasibility of a Cognitive Training Program Through Magic Tricks for Children With Attention Deficit and Hyperactivity Disorder. A Second Clinical Trial in Community Settings

**DOI:** 10.3389/fpsyg.2021.649527

**Published:** 2021-04-01

**Authors:** Saray Bonete, Ángela Osuna, Clara Molinero, Inmaculada García-Font

**Affiliations:** Department of Psychology, Universidad Francisco de Vitoria, Madrid, Spain

**Keywords:** attention deficit hyperactivity disorder, MAGNITIVE program, cognitive training program, magic tricks, effectiveness, feasibility

## Abstract

Previous studies have explored the impact of magic tricks on different basic cognitive processes yet there is a need of examining effectiveness of a cognitive training program through magic tricks for children with attention deficit hyperactivity disorder (ADHD). The present study examines the effectiveness and feasibility of the MAGNITIVE program, a manualized intervention for cognitive training through the learning of magic tricks. A total of 11 children with ADHD (from 8 to 12 years) participated in separated groups of two different community settings (hospital center and school), and were assessed at pre-treatment, post-treatment, and a 3-month later follow-up in different tasks involving processing speed, sustained attention, selective attention, and mental flexibility. Using non parametric statistical analyses and Reliable Change Index, the results showed that these children receiving MAGNITIVE particularly improved their performance in sustained attention, shifting attention, and mental flexibility, changes were also observed in processing speed performance yet further research is needed in terms of selective attention and inhibition, given the great individual differences within this sample. Changes were maintained when the program was finished. In terms of viability, the study proved a good treatment integrity in different contexts (hospital and school setting), adherence to the curriculum (attendance and some practice at home), and high levels of engagement satisfaction. In this second clinical trial, MAGNITIVE program appears to be a feasible training program for children with ADHD, as an alternative for medication when possible.

## Introduction

Attention deficit hyperactivity disorder (ADHD) is a neurodevelopmental disorder whose principal symptoms are hyperactivity, inattention, and impulsivity [American Psychiatric Association (APA), 2013]. Considered an impairment of the executive functions (Barkley, 1997), ADHD affects a series of cognitive processes related to self-regulation, task organization, planning, working memory, cognitive flexibility, time and space organization, emotional regulation (Pineda et al., 1998; Willcutt et al., 2005a,b; Colomer et al., 2017; Fabio et al., 2018), automatic and controlled processes (Capri et al., 2020), and other alterations of basic functions such as processing speed (Woods et al., 2002; Willcutt et al., 2005a). This is a persistent condition that can cause significant personal, familial, social, and educational difficulties (DuPaul et al., 2001; Rodríguez-Salinas et al., 2006; Deault, 2010; Pinho et al., 2017; Velõ et al., 2019).

Several studies have been conducted in recent years into the effectiveness of non-pharmacological treatments for ADHD (Pelham and Fabiano, 2008; Sonuga-Barke et al., 2013; Richardson et al., 2015; Watson et al., 2015; Scionti et al., 2020; Shrestha et al., 2020; Veloso et al., 2020). These non-pharmacological strategies include behavior modification techniques and cognitive behavioral therapy (Fabiano et al., 2009); cognitive training (Tamm et al., 2010; Shuai et al., 2017); training in self-instruction and techniques to enhance the capacity to inhibit responses (Meichenbaum and Goodman, 1971) including computer-based interventions (Martinovic et al., 2016; Rossignoli-Palomeque et al., 2018), training in problem solving (Bransford and Stein, 1993), neurofeedback (Zuberer et al., 2018; Cueli et al., 2019), training in social skills (Sheridan et al., 1996; Storebo et al., 2012), peer intervention (Cordier et al., 2018), and training in organizational skills (Langberg et al., 2008); and psycho-educational strategies and instruction of parents and teachers in cognitive behavioral techniques (Miranda et al., 2002; Pelham and Fabiano, 2008; Montoya et al., 2011; Rimestad et al., 2019); within the multi-modal approach, of particular importance are interventions focused on producing changes in neuro-psychological functions (Pistoia et al., 2004).

Studies have found positive results from training in basic cognitive processes, leading to general improvement in executive functions (Johnstone et al., 2012; Dias and Seabra, 2016; Lambez et al., 2020; Veloso et al., 2020). Furthermore, studies show the benefit of combining training in executive functions with other therapeutic strategies such as training in self-instruction (Meichenbaum and Goodman, 1971), modeling, and self-reinforcement (and other behavior modification techniques), for the improvement of sustained attention, selective attention, planning, social skills, academic performance, and the principal symptomology of ADHD (Miranda et al., 2002; Arco Tirado et al., 2004; Pérez, 2007; Ramalho et al., 2011; Sun, 2017). Studies have analyzed the effects of computerized cognitive training programs by itself or as supplementary interventions, finding positive outcomes in Intelligence Quotient (IQ) test (Fabio et al., 2019), working memory (Dovis et al., 2015; Johnstone et al., 2017; Passarotti et al. 2020), and symptoms of ADHD (Prins et al., 2013).

Evidence shows that learning by games and practice using play activities among children can facilitate the internalization of learned strategies and increase motivation (Pérez, 2007; Blasco-Fontecilla et al., 2015; Martinovic et al., 2016). Practicing physical exercise appears as one of the most effective non-pharmacological interventions in order to reduce some of ADHD cognitive symptoms (Lambez et al., 2020). One play activity that has sparked particular interest recently is magic (Kuhn et al., 2008; Bagienski and Kuhn, 2019). It has been demonstrated that learning magic tricks requires self-control, concentration, selective attention, the capacity for sequencing, planning, problem solving, an adequate level of working memory, and constant practice; skills which all involve executive functions (Rensink and Kuhn, 2014). A number of studies have examined the impact of magic tricks on perception (Rensink, 2010), control of visual attention (Kuhn et al., 2014; Rensink, 2015), critical thinking and creativity (Wiseman and Watt, 2020), reasoning and cognitive abilities (Wiseman and Watt, 2018), and the underlying mechanisms of memory and mnesic distortions of the neural bases of causality (Parris et al., 2009; Rensink and Kuhn, 2014). Equally, practicing magic has proved beneficial in the development of motor skills, imagination, problem-solving, and self-esteem (Spencer, 2012; Green et al., 2013; Harte and Spencer, 2014), interpersonal communication, and resilience, although studies finding these benefits did not use deep statistical analysis but were based on the experience of therapists or patients (Wiseman and Watt, 2018; Bagienski and Kuhn, 2019).

MAGNITIVE, described in detail elsewhere (Bonete et al., un-published), is a manualized program for cognitive training through the learning of magic tricks. It was developed specifically as a non-pharmacological alternative for children with ADHD.

There is currently no manualized program for cognitive training through magic whose effectiveness has been studied on children with ADHD. A study by Spencer (2012) on the benefits of incorporating an organized and systematic set of simple magic tricks into academic curricula found improvements in behavior, fine motor skills, self-esteem, and socialization as well as improved planning and sequencing skills. However, the study did not analyze changes before and after the intervention with psychological tests of cognitive functions.

The first MAGNITIVE pilot study was conducted with seven children between the ages of 8 and 12 diagnosed with ADHD and who were not receiving pharmacological treatment. It consisted of a 10-week session conducted by a therapist who was an expert magician. The results showed significant improvement in processing speed and selective attention as a group. Individually, three of the seven children improved their sustained attention and two of them improved their information processing speed (Bonete et al., 2016). After this implementation, improvements were made in the content and application of the program, based on the input and feedback of the therapist-magician and the participants.

The principal aim of the present study is to examine the effectiveness and viability (Kraemer and Kupfer, 2005) of the MAGNITIVE program, designed to develop the cognitive abilities of children with ADHD. This second clinical trial was conceived based on the model proposed by Smith et al. (2007) for addressing methodological challenges in research on psychosocial interventions. At a second phase, manualization of the intervention is fundamental to standardize it and make the manual available to other professionals (Smith et al., 2007). The study set out to examine the preliminary effectiveness of the program and confirm its viability and applicability in a second sample to explore the viability to be delivered as planned across settings, following the recommended steps for the evaluation of psychosocial intervention programs (Smith et al., 2007; Leon et al., 2011). The effectiveness of MAGNITIVE was determined based on changes Pre-Post participation in cognitive tasks that are maintained at the follow-up. The hypothesis proposed here is that cognitive training through magic can improve the performance of tasks involving the evaluation of cognitive processes and executive functions (processing speed, sustained attention, selective attention, mental flexibility, and planning ability). The viability was determined through an analysis of the fidelity of the therapist to the program (treatment integrity), adherence to the treatment, and the satisfaction of the participants (Pavuluri et al., 2004; White et al., 2012).

## Materials and Methods

### Participants

**Figure 1:**
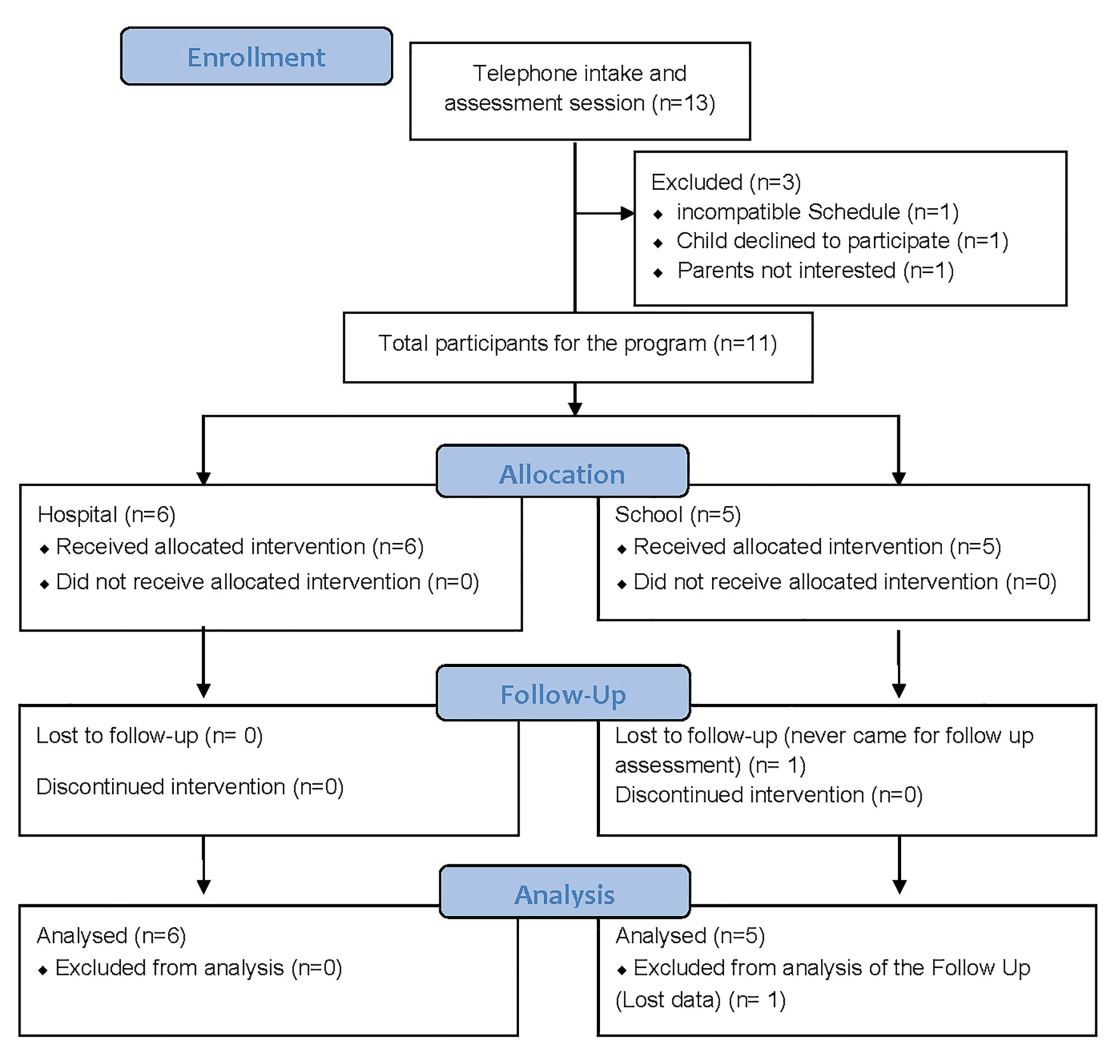
CONSORT flow chart of participants.

The final sample consisted of 11 children diagnosed with ADHD from the pediatric ward of the El Escorial Hospital (eight boys and three girls) organized in two community settings (hospital center and school). Participation was voluntary. The participants were selected based on the following inclusion criteria: (1) between 8 and 12 years of age; (2) diagnosed with ADHD by their pediatrician and who had high scores at the EDAH Questionnaire (assessment of ADHD test; Farré i Riba and Narbona García, 2013); (3) have a global IQ score on the Kaufman Brief Intelligence Test (K-BIT; Kaufman and Kaufman, 1997); (4) not be taking medication at the moment of the study; and (5) not suffer from any other serious psychiatric pathologies. Table 1 shows the descriptive characteristics of the sample including scores. Two of the children had dyslexia (5 and 6), but it was decided to include them in both the program and the data analysis to achieve a more representative sample and explore the effect of the program on children who are often diagnosed with this comorbidity (Germanò et al., 2010). See Figure 1 of the flow chart.

**Table 1:** Mean symptom severity characteristics of attention deficit hyperactivity disorder (ADHD) group.

Variable	*N*	*Mean*	*SD*
Age	11	9.82	1.40
Total IQ KBIT	11	91.91	6.98
EDAH – Hyperactivity (Parents)	11	77.82	4.42
EDAH – Attention deficit (Parents)	11	78	14.65
EDAH – Behavioral disorder (Parents)	11	74.09	11.14
EDAH – Hyperactivity and attention deficit (Parents)	11	81	13.65
EDAH – Hyperactivity (Teacher)	9	75	13.92
EDAH – Attention deficit (Teacher)	9	82.89	14.01
EDAH – Behavioral disorder (Teacher)	9	66.11	10.54
EDAH – Hyperactivity and attention deficit (Teacher)	9	80.44	12.17

IQ KBIT, Intelligence Quotient through Kaufman Brief Intelligence Test; EDAH, EDAH scales for ADHD assessment through parents’ and teacher’s test version (centile scores).

### Procedure

After receiving the approval of the ethics committee, researchers got in touch with parents of patients who met the inclusion criteria and were interested in participating in a non-pharmacological treatment program. The parents were invited to a group information session about the MAGNITIVE program and the objectives of the study. Those interested in the voluntary participation of their children, provided their informed consent and contact details. Appointments were scheduled for an individual pre-treatment psychological assessment of the child and at least one of the parents was also interviewed (Pre). The parents were provided with a questionnaire for their child’s teachers. Parents and teachers reported EDAH scores as a supplementary measure for diagnostic confirmation. The final sample consisted of 11 children organized into two training groups of five and six participants. The treatment was held at the hospital for the first group (*n* = 5) and at a local school (*n* = 6) attended by the rest of the sample. A post-treatment assessment was conducted in the same week the program concluded (Post), as well as a follow-up evaluation 3 months later (Post-2) using the same tests. All assessments were performed by two research psychologists of the team. A psychotherapist, who is also an experienced magician, delivered the program with a facilitator who assisted with materials and coding integrity of the program.

### Intervention: MAGNITIVE

The manualized MAGNITIVE program (Bonete et al., un-published) was designed for school children (in primary education) diagnosed with ADHD and implemented in small groups (5–6 participants). The program consisted of 10 group-training sessions of 60–75 min, including a break. Each session focused on learning a magic trick or developing the prerequisite skills necessary to perform the trick. The program followed a standardized order, teaching magic tricks of increasing difficulty as the program advanced interspersed with handicraft sessions to create a magician’s costume. The program had dual objectives: to instruct the children to perform magic tricks and to train cognitive abilities through a play activity using various techniques, which scientific studies have associated with improved executive functions (MTA Cooperative Group, 1999; Espinosa, 2006; Ives, 2006; Orjales, 2007; Fabiano et al., 2009). The training was conceived to be conducted by a therapist who was also a professional magician.

#### Integrity of the Treatment/Therapist Fidelity to the Program

This refers to the degree to which the therapist delivered treatment as intended. A treatment integrity checklist was used to examine the following of the schedule and tasks by the therapist while delivering the program. During each session, the facilitator recorded how many of the session’s objectives were completed. Following Pavuluri et al. (2004), integrity was assessed as a percentage (percentage of delivered treatment components/number of planned components for that session*100), considering 80% set as a minimally acceptable level.

#### Adherence to the Program/Subject Involvement

A record was kept of the participants’ attendance and the number of hours they practiced at home with the help of a register. Before the beginning of the program, parents were provided with a register on which they were asked to note daily, the amount of time (in minutes) their children practiced magic tricks at home. The parents were not specifically asked to encourage their children to practice. The register was submitted at the end of the program. The study included a weekly calculation of the practice time.

#### Subject Satisfaction

In the final evaluation at the end of the program, the children and parents were asked about their satisfaction with the program, using a Likert scale from 1 (not satisfied) to 5 (completely satisfied), evaluating the effect of the program in various aspects: knowledge of magic, entertainment, and use of magic, following of instructions, organization, memory, shifting attention, following the norms and personal comments. The scores varied from 10 to 50. Two items concerning social validation of the program were included. The questionnaire was also submitted to the tutor or teacher of reference of the student.

#### Follow-Up Questionnaire Three Months Later

A follow-up session was held 3 months after the program with parents and children to evaluate their performance in psychological tests used to determine the effectiveness of the program.

### Instruments

#### Five Digits Test

This test (FDT; Sedó, 2007) measures processing speed (reaction time) principally through the components of *Reading* (Reading-FDT) and *Counting* (Counting-FDT); inhibition through the component of *Choosing* (Choosing-FDT) and cognitive flexibility/alternating attention through the component *Shifting* (Shifting-FDT). The reliability coefficients for Spanish samples *α* = 0.94 (Reading-FDT), *α* = 0.92 (Counting-FDT), *α* = 0.86 (Choosing-FDT), and *α* = 0.90 (Shifting-FDT). The test is very effective in neuropsychological analysis and suggests high intercorrelations between the four components in the test, between 0.66 and 0.93. The results are presented as centile scores, with higher scores indicating better performance.

#### D2 Test of Attention

This test (D2; Brickenkamp and Seisdedos Cubero, 2012) measures sustained attention using a *concentration performance index* (Concentration-D2), selective attention using an *omission errors index* (Omission-D2), degree of impulsivity using a *commission errors index* (Commission-D2), and processing speed. This provides a score for *Total test effectiveness* [taken from the *total n° responses* (number of characters correctly processed) less the omission and commission errors); the *total number of correct answers*, as a measure of the work performed as well as the *Attention variation index* (Variation-D2), which evaluates the stability of work overtime. In most studies, the results of the D2 test have proven highly reliable (*r* > 0.90). The results are presented as centile scores, with higher scores indicating better performance (Jiménez et al., 2012).

#### Coding and Digit Span Subtests of the Wechsler Intelligence Scale for Children

The *Coding* subtest (Coding-WISC; Wechsler, 2005) evaluated processing speed and sustained attention. *Digit span* (Digit Span-WISC; Wechsler, 2005) evaluates attention, short-term memory, and working memory. The results of these subtests are on a scaled score and have an average reliability of 0.84.

#### Battery of Neuropsychological Assessment for Executive Function in Children

This is a comprehensive evaluation (ENFEN; Portellano et al., 2011) of the global development of children between the ages of 6 and 12. It consists of six tasks which measure distinct or simultaneous cognitive processes. The *Phonological Fluency* task evaluates phonological fluency; the *Semantic Fluency* tasks evaluate semantic fluency. The *Gray Path* task evaluates processing speed. The *Color Path* task evaluates mental flexibility. The *Rings* task evaluates thought organization, anticipation, sequencing, and working memory; the final task, *Interference* evaluates working memory, prospective memory, and resistance to interference – inhibition. For the study, raw scores were presented, given that the manual only offers sten scores. Higher scores indicate better performance in all tests except the Rings task in which scores are reversed. Different studies show evidence of the suitability of the instrument (Navarro-Soria et al., 2020). Table 2 summarizes the characteristics of the measures used.

**Table 2:** Summary of labeling of the subtests, cognitive skills measured, type and range of score.

Cognitive skills	Subtest/task (score scale type)	Range of score for 8–12 year children	
		Min	Max
Processing speed	Reading-FDT (CS)	>43	<14
	Counting-FDT (CS)	>59	<17
	Effectiveness-D2 (DS)	0–94	430–658
	Total Responses-D2 (DS)	0–121	442–658
	Coding-WISC (SS)	0–11	85–119
	Gray Path-ENFEN (DS)	0	>55
Sustained attention	Concentration-D2 (DS)	0–6	174–299
	Coding-WISC (SS)		
Selective attention	Omissions-D2 (DS)	>33	0
Stability of attention	Attention variation Index	0–4	33–47
Shifting attention/Cognitive flexibility	Shifting-FDT (CS)	>88	<28
	Color Path-ENFEN (DS)	0	>33
	Digit Span-WISC (SS)	0–2	26–32
Inhibition/Impulsivity	Choosing-FDT(CS)	>96	<31
	Commission-D2 (DS)	>38	0
	Interference-ENFEN (DS)	0	>148
Mental flexibility and others	Color Path-ENFEN (DS)	0	>33
	Rings-ENFEN (DS)	>326	<96
	Interference-ENFEN (DS)	0	>148
	Phonological Fluency-ENFEN (DS)	0	>21
	Semantic Fluency-ENFEN (DS)	0	>28

FDT, five digits test; D2, D2 test of attention; WISC, Wechsler intelligence scale for children; ENFEN, battery of neuropsychological assessment for executive function in children; DS, direct score; CS, centile score; and SS, scaled score.

### Statistical Analysis

The study consisted of an open trial of a manualized program for performing magic tricks in groups. Given that the objective was to assess the viability of the program in a communal (non-artificial) environment, the sample was selected by convenience (Lucas, 2013).

To evaluate behavioral changes after the treatment, an initial non-parametric analysis was conducted to determine if there were any significant differences between the three moments of evaluation as a whole period: Pre-Post-Post2, by means of the Friedman *X*^2^ test (*n* = 10). Non-parametric tests were used due to the size of the sample. Secondly, non-parametric *post hoc* tests were conducted to identify exactly when these differences occurred (Pre-Post *n* = 11, Post-Post 2 *n* = 10), using Wilcoxon Z-test. Effect size, using Kendall’s W, and statistical power were reported for each significant difference.

The clinical significance or magnitude of the individual change was also calculated using the Reliable Change Index (RCI; Jacobson and Truax, 1991), according to which a change in (direct) scores greater than 1.96 SD is considered statistically and clinically significant in each participant.

The feasibility of the program was determined by evidence that the treatment is acceptable for the target population and may be applied reliably based on the dropout rate, attendance rates, the hours of practice at home, the participant satisfaction questionnaires, and the fidelity of the therapist to the program.

## Results

### Changes After Treatment and Follow-Up Assessment

A comparison of the average results of the Pre, Post, and Post 2 (3 months after the end of the program) tests showed statistically significant changes at the three moments of evaluation (Pre-Post-Post2) with moderate effect sizes (all over 0.40) in: Coding-WISC, Color Path-Battery of Neuropsychological Assessment for Executive Function in Children (ENFEN), Rings-ENFEN, Total Responses-D2 and Correct Responses-D2, Omission-D2, Effectiveness-D2, Choosing-FDT, and Shifting-FDT. *Post hoc* tests were also conducted using the Wilcoxon *Z*-test for a two-by-two comparison of the three moments of evaluation: Pre-Post and Post-Post2; to see if changes appear after the program, and last 3 months after. Table 3 shows the significant differences, effect size, and power.

**Table 3:** Descriptive statistics and non-parametric differences in repeated tasks Pre-Post-Post 2 (*X*^2^ Friedman), *post hoc* tests (Wilcoxon *Z*), and effect size.

Variable (unit of measure)	Pre	Post	Post 2	*X*^2^_(*gl* = 2)_	*W*	Statistical power (1-β)	*Post hoc*	
	M (*SD*)	M (*SD*)	M (*SD*)				Pre-Post *Z* (r)	Post-Post 2 *Z* (r)
**WISC (SS)**								
Digit Span	6.8 (2.20)	7.70 (2.21)	6.60 (2.87)	2.24	-	-	−1.62	−1.44
Coding	7.30 (2.06)	9.10 (2.81)	8.90 (2.84)	8.32^*^	0.42	0.72	−2.53^*^(0.80)	−0.34
**ENFEN (DS)**								
Phonological Fluency	9 (2.92)	9.89 (3.41)	9.89 (3.48)	0.23	-	-	−1.13	−0.60
Semantic Fluency	15.89 (6.17)	16.11 (3.72)	16.00 (5.09)	0.18	-	-	−0.04	0
Gray Path	17.84 (4.79)	22.30 (7.91)	21.74 (6.00)	2.60	-	-	−2.31^*^(0.73)	−0.51
Color Path	8.92 (3.99)	11.76 (5.07)	11.38 (7.18)	8.60^*^	0.43	0.72	−1.87	−0. 25
Rings	235.50 (48.10)	178.60 (25.11)	165.80 (33.15)	14.60^**^	0.73	0.93	−2.76^**^(0.87)	−1.58
Interference	60.37 (17.56)	63.62 (17.03)	59.89 (29.21)	0.22	-	-	−1.25	−0.06
**D2 (CS)**								
Total Responses	42.10 (24.44)	54.30 (29.14)	72.50 (29.07)	13.03^**^	0.65	0.91	−2.55^*^(0.81)	−1.96^*^(0.62)
Correct Responses	40.10 (22.66)	51.50 (22.86)	71.90 (22.61)	13.47^**^	0.67	0.91	−2.14^*^(0.68)	−2.52
Omission	25.90 (18.20)	39.80 (33.28)	50.90 (28.64)	8.40^*^	0.42	0.50	−1.60	−1.37
Commission	36.60 (26.52)	41.20 (27.65)	42.00 (27.51)	0.68	-	-	−0.59	−0.15
Total Effectiveness	40.10 (23.74)	53.80 (28.01)	71.60 (29.99)	15.17^**^	0.76	0.94	−2.68^**^(0.85)	−1.96^*^(0.62)
Concentration	35.60 (20.55)	49.80 (24.00)	65.80 (36.82)	11.11^**^	0.56	0.85	−2.56^*^(0.81)	−1.89
Variation	36.40 (25.85)	56.00 (26.01)	50.00 (19.86)	1.37	-	-	−1.96^*^(0.62)	−0.71
**FDT (CS)**								
Reading	13.70 (15.94)	17.30 (22.35)	8.70 (10.55)	0.64	-	-	−0.93	−1.36
Counting	14.60 (23.39)	27.40 (35.17)	17.40 (24.98)	5.64	-	-	−2.38^*^(0.75)	−1.18
Choosing	14.50 (22.86)	27.00 (32.48)	32.00 (34.79)	8.58^*^	0.43	0.75	−2.03^*^(0.64)	−0.49
Shifting	8.80 (13.48)	28.20 (28.06)	28.20 (26.80)	10.56^**^	0.53	0.84	−2.52^*^(0.80)	0

M, mean; SD, standard deviation; DS, direct score; CS, centile score; SS, scaled score; FDT, five digits test; D2, D2 test of attention; WISC, Wechsler Intelligence scale for children; ENFEN, battery of neuropsychological assessment for executive function in children; and *r*, *post hoc*effect size when applicable.

* *p*< 0.05;

** *p*< 0.01.

In terms of **processing speed**, the analysis showed significant changes in the three moments as a whole (Pre-Post-Post 2) in Effectiveness-D2 and Total Responses-D2, and changes exclusively in Pre-Post in Counting-FDT and Gray Path-ENFEN. It did not show changes in Reading-FDT nor Coding-WISC.

Regarding attention, there were several sources providing information. As regards **sustained attention**, Concentration-D2 and Coding-WISC both show statistically significant improvements at the three moments, and in Pre-Post, changes remain in Post 2. In **selective attention**, Omissions-D2 showed significant differences at the three moments. As for **shifting attention**, the Shifting-FDT test showed statistically significant changes at the three moments, and specifically in Pre-Post; those changes remain in Post-2. Likewise, the change already mentioned at the three moments in Color Path-ENFEN was an evidence of this improvement. The **attention variation index**showed statistically significant changes in all three moments.

With respect to **inhibition/impulsivity**, statistically significant changes were observed in Choosing-FDT both at the three moments (Pre-Post-Post 2) and at the *post hoc*test (Pre-Post). This was not the case in the calculation of Commission-D2 nor in Interference-ENFEN.

Regarding **mental flexibility**(working memory tasks and short-term memory), there were statistically significant changes at the three moments in Color Path-ENFEN and Ring-ENFEN (*p*< 0.01), thus confirming, in *post hoc*tests, a Pre-Post change that remains in Post-2 (where there were no significant changes in the absence of the program). On the other hand, statistically significant changes were not observed in Interference-ENFEN nor in the tasks associated with Phonological fluency-ENFEN and Semantic Fluency-ENFEN.

### Clinically Significant Changes

Table 4shows the RCI scores for the various tasks. Participants 2, 3, 7, and 11 showed statistically significant clinical changes in the greatest number of variables. The tests or tasks which showed the greatest statistically significant change were Correct Responses-D2, Comission-D2; three of these children also showed improvements in Color Path-ENFEN (2 and 3), Omission-D2 (2, 7, and 11), and Rings-FDT (2, 3, and 7). Another three showed improvement in Semantic Fluency-ENFEN (2, 3, and 6) and Concentration-D2 (2, 7, and 10). Additionally, participants 2 and 7 improved in Phonological Fluency-ENFEN while participants 2 and 4 improved in Digit Span-WISC.

**Table 4:** Reliable change index (RCI) post-pre program for each participant.

Variable	1	2	3	4	5	6	7	8	9	10	11
**WISC (SS)**											
Digit Span	+			+							
Coding										−	
**ENFEN (DS)**											
Phonological Fluency		+					+				
Semantic Fluency		+	+			+			−		
Gray Path											
Color Path		+	+								+
Rings							−				
Interference	−							−	−		
**D2 (CS)**											
Total Responses		−									
Correct Responses		+	+				+				+
Omission		+					+				+
Commission		+	+	−			+			−	+
Total Effectiveness		−					+				
Concentration		+					+			+	
Variation		+				+				+	+
**FDT (CS)**											
Reading		+	+	−			+				
Counting						−					
Choosing		−	−		−	−				+	
Shifting	−					−					

RCI, reliable change indexes; (+) = RCI > 1.96 SD, significant clinical improvement; and (−) = RCI significantly worse performance.

It is also interesting to highlight those tasks where individuals showed clinically significant changes with lower scores, specifically in Choice-FDT (2, 3, 5, and 6), and Interference-ENFEN (1, 8, and 9). However, two participants showed worse results in the Post for Shifting-FDT (1 and 6) and Comission-D2 (4 and 10). Surprisingly, participant 2, one of those showing most improvement in all tests, had the lower score in Total Responses-D2, Total Effectiveness-D2, and Choice-FDT.

Regarding the two participants with dyslexia, participant 6 showed worse results in Shifting-FDT and Choice-FDT, but improved Semantic Fluency-ENFEN, while participant 5 showed improvements in Rings-FDT, with statistically worse Post scores only in Choice-FDT.

In summary, changes in group-mean scores related to **processing speed**, were shown by clinically significant improvements in three of the 11 participants in Rings-FDT and one in Total Effectiveness-D2. As for attention, clinically significant improvements appeared in: (1) **selective attention**, as three participants showed significant improvements in Omissions-D2; (2) **sustained attention**, with improvement of three participants in Concentration-D2; and (3) **shifting attention**, not so clearly in this case, as three participants improved significantly at a clinical level for the Color Path-ENFEN and two obtained worse scores after the intervention in Shifting-FDT. However, attention **stability of attention**seemed to improve in the performance of four of the 11 participants (Variation-D2). In terms of **inhibition and impulsivity**, individual patterns were miscellaneous: only one participant improved at a clinical level in Choosing-FDT, whereas four others seemed to do it significantly worse, among them, the two participants diagnosed with dyslexia. Four participants performed significantly lower Commission-D2, but three performed clinically worse in Interference-ENFEN. Regarding the rest of executive functions, three children showed changes in Semantic Fluency-ENFEN and two in Phonological Fluency-ENFEN, but the tests did not allow these differences to be captured in mental flexibility or working memory.

### Feasibility: Integrity of the Treatment, Adherence to the Program, and Subject Satisfaction

The feasibility of the program was calculated based on the integrity of the application by the therapist-magician, the drop-out rate, and the adherence of the participants to the treatment (attendance rate), hours of practice registered by parents and the satisfaction questionnaire completed by the children, parents, and teachers. It was hoped that the changes would persist 3 months later.

Regarding the integrity of the treatment over the course of 10 sessions in two groups, overall integrity was 85.7%. For the hospital group, integrity was 91.1%, while at the school integrity was 81.3%. An analysis of the sessions showed than none fell below the 80% cut-off for acceptable treatment integrity. The sessions were conducted within the allotted time (60–75 min), although time was always tighter in the school group. The qualitative evaluation of the therapist indicated greater difficulty in maintaining the pace of sessions imparted at the school as an extracurricular activity.

Adherence of the participants to the program included participation in the weekly sessions and hours of practice at home, recorded by the parents. All the participants remained in the program to the end, attending all 10 sessions. The overall rate of attendance (a total of 110 sessions, two groups by 10 sessions) for the 11 participants was 96.36%, seven children attended all the sessions and four children missed only one session. Considering the overall baseline attendance criteria of 70%, this was considered an excellent result for this indicator (Pavuluri et al., 2004).

With reference to practice at home, four of the 11 children practiced between the sessions at home, observed and recorded by the parents. Of the children who attended sessions at the hospital, only one practiced at home between sessions from 10 to 25 min per week during the first six sessions, stopping practice thereafter. In the school group, three children practiced at home, from 3 to 90 min per week. The variation between the three was enormous. One participant only practiced at home until session 5, while another two managed to dedicate significant amounts of time throughout the course of the program.

Regarding the satisfaction questionnaire, of the 10 items dealing with changes in daily life, 69.1% of all responses by the children were 4 or 5 (somewhat or very much), while in the case of parents, similar responses were given 26.4% of the time. In the qualitative analysis, nine children considered the ability *to do magic tricks after the program*, as a significant change, which was confirmed by parents of all the children and *having more friends*after participating in the program, confirmed by the parents of three children. Eight of the children marked 4 (somewhat) for *it is easier to follow instruction than before*, and they *feel able to stay seated more time*(conformed by the parents of three children), and seven indicated that they *learned to be better organized, have a better memory, and can better follow norms of behavior*(coinciding with the response from parents in only two cases). Notably, nine of the children affirmed that they had greatly enjoyed the program, a highly positive result for the therapist-magician. One participant commented on the benefit of now having something to do when bored. Two of the children mentioned the importance of making new friends. Four of the parents commented that their children were highly motivated to attend the sessions and in two cases improvements were reported in academic performance. As for teachers, the three questionnaires which were returned provided little useful information. All the children and parents affirmed that they would recommend the program to others and that the program should receive financial support from social services in order to be available to more children.

## Discussion

The aim of the present study was to evaluate in a second clinical trial in community settings, after conducting a previous pilot study, the effectiveness and feasibility of MAGNITIVE program for groups of children with ADHD; a program based in the implementation of self-instructions and other cognitive-behavioral techniques through the teaching of magic tricks. Under two different types of analysis of treatment effectiveness, findings reveal a significant improvement in these participants regarding cognitive abilities. On the one hand, based on changes in participants’ performance (group mean scores) of different cognitive tasks measuring processing speed, attention processes (shifting attention, sustained attention, selective attention, stability of variation in attention, and inhibition), mental flexibility and other executive functions such as phonological and semantic fluency. And secondly, as a complementary analysis, RCI changes for each participant were calculated between two moments. Taking the two methodologies into account, this training program particularly increased sustained attention, shifting attention, and mental flexibility of the participants. Processing speed performance significantly changed when the group mean scores were taken. Meanwhile, the impact of this training should be further studied in randomized clinical trials and larger samples, especially in order to clarify its effects in terms of selective attention and inhibition, given the great individual differences within this sample.

In general terms, statistical analysis showed that in all cases where there were significant changes at the three assessed moments (Pre-Post-Post2), the *post hoc*confirmed this improvement in Pre-Post, and sometimes also in Post-Post2. It was never the case that for a statistically significant change at the three moments (Pre-Post-Post2), the *post hoc*tests revealed that the change was significant only Post-Post2 without previous significant changes at Pre-Post. Therefore, changes appeared always in the presence of the program, and in any case, could be maintained when the program is finished but, improvements do not occur alone, in the absence of the program. Furthermore, under no circumstances the acquired skills in the presence of the program worsened 3 months later (after the program was finished).

In connection with the improvements in processing speed and sustained attention, the results obtained were consistent with the results in the first pilot study of MAGNITIVE program (Bonete et al., 2016), and with previous studies based on cognitive-behavioral interventions that emphasize these cognitive skills (Minder et al., 2019), self-instructions (Arco Tirado et al., 2004; Ramalho et al., 2011), and studies examining the effects of different play activities (Blasco-Fontecilla et al., 2015; Schmitt et al., 2018).

No previous interventions were found, reporting improvement in processes such as organization, sequencing, and anticipation with a cognitive-behavioral focus (Shuai et al., 2017). Some studies based on computerized cognitive training programs are promising (Johnstone et al., 2012; Dovis et al., 2015; Minder et al., 2019; Passarotti et al., 2020) although there is also evidence of differences among interventions to find generalized improvement of ADHD symptomatology (Smith et al., 2020). The present study does show changes in tasks that involve mental flexibility (Color Path-ENFEN and Rings-ENFEN), needed for planning. It is interesting to highlight how they are processes that the empirical evidence shows as specially affected in people with ADHD (Pineda et al., 1998; Woods et al., 2002; Willcutt et al., 2005a) and they are also key processes in the mastery of magic (Rensink and Kuhn, 2014). The work by Spencer (2012), that used magic inside the academic curriculum, pointed at improvements in planning and sequencing. Our study constitutes a relevant empirical evidence as there are few previous studies incorporating magic on their cognitive training program for children with ADHD (Harte and Spencer, 2014; Wiseman and Watt, 2018; Bagienski and Kuhn, 2019).

In relation to inhibition/impulsivity, there was a statistically significant change in one of the tasks, and clinically significant improvements in some of the participants in different tests associated with inhibition, although other participants showed a significantly worse performance. This cognitive ability seems to be the least benefitted from the program, compared to the rest of abilities. It is worth considering whether the learning of magic tricks does not help in training inhibitory control (Diamond and Ling, 2016) or whether it is the tasks selected to assess the cognitive processes involved that fail to show the impact of this learning on impulsivity management (Arco Tirado et al., 2004; Pérez, 2007; Mattfeld et al., 2016).

The results in terms of feasibility are promising. Until now, few evidence-based interventions focused on improving cognitive abilities through play activities (Spencer, 2012; Blasco-Fontecilla et al., 2015), which makes these findings unique and highly relevant. Firstly, this study proves that it can be applied consistently (treatment integrity) and participants in general showed adherence to the curriculum (attendance and practice at home) and high levels of engagement satisfaction. The study also made it possible to analyze the program’s effectiveness, as positive changes could be found in different contexts (hospital or health center and school setting) which is highly valuable in testing treatment effectiveness (Smith et al., 2007; Rosa et al., 2017). Interestingly, the engagement rate in the program and the high attendance level show how the use of play activities for cognitive training becomes a source of greater motivation to create change. Besides, the content in itself makes easier its introduction in school curriculum or extracurricular education offered by schools (Dias and Seabra, 2016). This would facilitate the incorporation of many children with these needs into de program (Pérez, 2007).

In conclusion, following the step-wise model for validating and disseminating interventions (Smith et al., 2007; Leon et al., 2011), MAGNITIVE as manualized program, showed in a second clinical trial improvements in Pre-Post that were maintained 3 months after the end of magic initiation training. This means that the program was effective (Kraemer and Kupfer, 2005) for training without having to resort to medication. Even more, some gains in attention were not only maintained but keep improving in the absence of the program (see Table 3).

Previous studies with play activities such as chess games (Blasco-Fontecilla et al., 2015) and computer games (Pérez, 2007; Prins et al., 2013; Dovis et al., 2015; Martinovic et al., 2016; Johnstone et al., 2017) highlight the potential of these contents in keeping up motivation while training abilities and promoting generalization, which is so difficult to obtain (Espinosa, 2006; Cortese et al., 2015; Meyer et al., 2020; Passarotti et al., 2020). Mastering magic requires constant practice from the interested person, and as this field provides endless possibilities, it promotes creativity. Participants are expected to internalize these lessons and keep applying them in various contexts of their life, thus promoting generalization. This natural tendency in the children to show the tricks in every stage they have could become an advantage, comparing to other attempts to promote generalization (like proposing different virtual reality settings). Children enjoy performing their new tricks in front of friends, family, teachers, etc.; which is a powerful reinforcement. The next step would be to examine whether the program could create a greater impact if it had a broader reach (Bagienski and Kuhn, 2019; Spencer et al., 2019), or whether it could spark the child’s motivation to strengthen his/her skills for different tricks so that it could extend in time the benefits of the program with no need for another intervention.

Among the limitations of the study, the sample recruited was small. Larger samples with a control group of comparison may increase statistical power. The lack of a wait list group or comparison with another training package prevents the direct attribution of the changes to MAGNITIVE program against other unspecified aspects of the program. There is the fact that the same psychotherapist carried out the whole program with two groups (11 children in total), which also reduces the generalization of results. Another limitation refers to the chosen tests to examine changes in inhibition and impulsivity among other cognitive abilities. Practice at home was not requested consistently nor registered in a reliable manner. It would be interesting to analyze the program’s effects if the package includes regulated home tasks within the program or psychoeducational intervention to parents (Montoya et al., 2011). In addition, an impact analysis of the program on participants’ interpersonal relations (Ison and Morelato, 2002; Bonete and Molinero, 2016) and on self-esteem (Spencer, 2012; Green et al., 2013) is lacking. Mastery in magic offers the children a resource for improving social interactions and promotes acceptance and integration in activities with peers. ADHD diagnosis implies certain behaviors that hinder interaction with other children and is associated with receiving constant criticism due to their behavior (Storebo et al., 2019; Willis et al., 2019). Magic tricks are an opportunity for them to receive positive attention from peers and adults, providing them with a social reinforcement for their skill, thus transforming their behavior in a source of pleasant moments in the group (instead of perceiving themselves as a cause of constant uneasiness).

In conclusion, this study shows the effectiveness in a second clinical trial of MAGNITIVE, a cognitive training through magic, whose objective is improving deficient cognitive abilities in a group of children with ADHD as part of a play activity. The study is draw from a manualized program as a starting point, which could spread over time, encouraging expertise in performing magic tricks. It has shown an adequate implementation feasibility both in school and hospital environments. MAGNITIVE program is ready to be validated with larger samples in multisite randomized clinical trials.

## Data Availability Statement

The raw data supporting the conclusions of this article will be made available by the authors, without undue reservation.

## Ethics Statement

This study was reviewed and approved by the Ethics Committee at Universidad Francisco de Vitoria. Written informed consent to participate in this study was provided by the participants’ legal guardian/next of kin.

## Author Contributions

SB and AO contributed to the conception and design of the work, assessed the children, and prepared the first draft of the manuscript with the assistance of IG-F. CM analyzed the data set, wrote the results section, and reviewed the paper draft. SB led the paper draft and revised the last version of the manuscript. All authors contributed to the article and approved the submitted version.

### Conflict of Interest

The authors declare that the research was conducted in the absence of any commercial or financial relationships that could be construed as a potential conflict of interest.
